# scRNA-seq revealed high stemness epithelial malignant cell clusters and prognostic models of lung adenocarcinoma

**DOI:** 10.1038/s41598-024-54135-4

**Published:** 2024-02-14

**Authors:** GuoYong Lin, ZhiSen Gao, Shun Wu, JianPing Zheng, XiangQiong Guo, XiaoHong Zheng, RunNan Chen

**Affiliations:** Department of Respiratory and Critical Illness Medicine, The First Hospital of Putian, Putian, 351100 China

**Keywords:** mRNAsi, Lung adenocarcinoma, Single-cell RNA-seq analysis, Immune microenvironment, WGCNA, Cancer, Computational biology and bioinformatics

## Abstract

Lung adenocarcinoma (LUAD) is one of the sole causes of death in lung cancer patients. This study combined with single-cell RNA-seq analysis to identify tumor stem-related prognostic models to predict the prognosis of lung adenocarcinoma, chemotherapy agents, and immunotherapy efficacy. mRNA expression-based stemness index (mRNAsi) was determined by One Class Linear Regression (OCLR). Differentially expressed genes (DEGs) were detected by limma package. Single-cell RNA-seq analysis in GSE123902 dataset was performed using Seurat package. Weighted Co-Expression Network Analysis (WGCNA) was built by rms package. Cell differentiation ability was determined by CytoTRACE. Cell communication analysis was performed by CellCall and CellChat package. Prognosis model was constructed by 10 machine learning and 101 combinations. Drug predictive analysis was conducted by pRRophetic package. Immune microenvironment landscape was determined by ESTIMATE, MCP-Counter, ssGSEA analysis. Tumor samples have higher mRNAsi, and the high mRNAsi group presents a worse prognosis. Turquoise module was highly correlated with mRNAsi in TCGA-LUAD dataset. scRNA analysis showed that 22 epithelial cell clusters were obtained, and higher CSCs malignant epithelial cells have more complex cellular communication with other cells and presented dedifferentiation phenomenon. Cellular senescence and Hippo signaling pathway are the major difference pathways between high- and low CSCs malignant epithelial cells. The pseudo-temporal analysis shows that cluster1, 2, high CSC epithelial cells, are concentrated at the end of the differentiation trajectory. Finally, 13 genes were obtained by intersecting genes in turquoise module, Top200 genes in hdWGCNA, DEGs in high- and low- mRNAsi group as well as DEGs in tumor samples vs. normal group. Among 101 prognostic models, average c-index (0.71) was highest in CoxBoost + RSF model. The high-risk group samples had immunosuppressive status, higher tumor malignancy and low benefit from immunotherapy. This work found that malignant tumors and malignant epithelial cells have high CSC characteristics, and identified a model that could predict the prognosis, immune microenvironment, and immunotherapy of LUAD, based on CSC-related genes. These results provided reference value for the clinical diagnosis and treatment of LUAD.

## Introduction

Lung cancer is a malignant tumor that originates in the mucous membranes or glands of the bronchus and is the leading cause of cancer-related death^1^. According to the latest data from the Global Cancer Survey 2020, there were 2,206,771 new cases of lung cancer and 1,796,144 deaths worldwide in 2020, making it the second most common cancer and the leading cause of cancer death^2^. Lung cancer consists of non-small cell lung cancer (NSCLC) and small cell lung cancer (SCLC), of which non-small cell lung cancer accounts for about 80–85% of lung cancer cases^3^, lung adenocarcinoma (LUAD) is the most common pathological subtype, accounting for about 50% of non-small cell lung cancer. It is characterized by complex mechanisms, strong aggressiveness, and poor prognosis^4,5^. In the last decade, there have been new advances in the treatment of LUAD, including surgical treatment, radiotherapy, chemotherapy and targeted combination therapy. However, due to the occult nature of the disease, most of the patients with LUAD were diagnosed at an advanced stage and could not be treated with surgery. However, after the use of other drugs for radiotherapy and chemotherapy, the prognosis of LUAD patients is still poor, and the 5-year survival rate is less than 20%^6^. Therefore, exploring the prognostic markers of LUAD has become the top priority of current scientific research.

Recent studies have shown that tumor growth may be driven by a small group of cells called cancer stem cells (CSCs). These cells may generate tumor host cells through self-renewal and multidirection differentiation, maintain tumor growth and heterogeneity, and are also called cancer initiating cells. CSCs have a pioneering immunosuppressive effect at the time of tumorigenesis, and gradually lose this ability during differentiation into astrocytes and oligodendrocytes. In addition, CSCs are believed to be extremely resistant to treatment, leading to multiple treatment failures, including immunotherapy. mRNA expression-based stemness index (mRNAsi), the stemness index of the transcriptome calculated by the OCLR algorithm, could be used to evaluate stemness. Higher mRNAsi scores, as reflected by histopathological grades, are associated with active biological processes in CSCs and with more differentiated tumors^7^.

Single-cell RNA sequencing (scRNA-seq) research has increasingly focused on the natural progression of cancer. A mouse model of esophageal squamous cell carcinoma (SQUamous cell carcinoma) induced by chemical carcinogen 4-nitroxylin 1-oxide (4-NQ0) was constructed to simulate the animal model of human esophageal carcinoma. The evolutionary trajectory of esophageal epithelial carcinoma from normal and precancerous lesions to invasive carcinoma was described in detail by single-cell transcriptome sequencing^8^. Single-cell analysis of precancerous lesion samples from gastric, pancreatic, and colorectal cancer showed that precancerous cells were also highly heterogeneous, with significant dynamic changes in cell composition and expression program^9,10^.

This study attempted to use single cell RNA analysis to identify malignant epithelial cells with high CSC, search for genes related to CSC, and construct prognostic models, hoping to predict patients' prognosis, immune status, and immunotherapy strategies.

## Results

### Difference analysis of mRNAsi in transcriptome datasets

**Figure 1:**
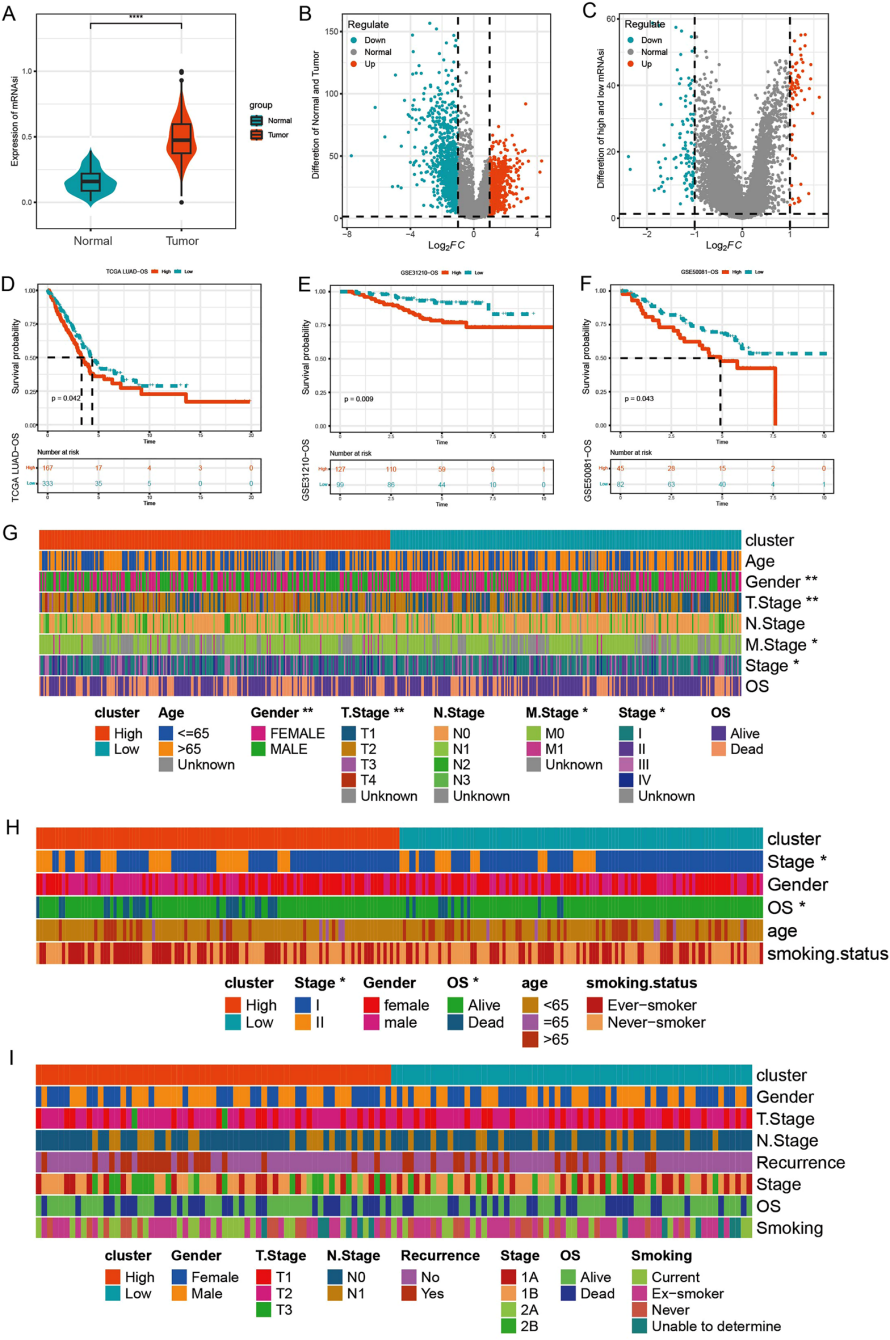
mRNAsi differences in the transcriptome of lung adenocarcinoma. (**A**) mRNAsi was higher in tumor samples than that in normal samples in TCGA dataset. (**B**) Volcano map of differentially expressed genes between tumor samples and normal samples in TCGA dataset. (**C**) Volcano map of differentially expressed genes between high- and low- mRNAsi tumor groups in TCGA dataset. (**D**–**F**) The survival times in high mRNAsi group was shorted than that in low mRNAsi group in TCGA dataset, GSE31210 dataset and GSE50081 dataset. (**G**–**I**) Differences in clinical features of high and low mRNAsi groups in TCGA dataset, GSE31210 dataset and GSE50081 dataset.

As described in method, mRNAsi of samples in TCGA-LUAD dataset were calculated, and higher mRNAsi in tumor samples were observed than that in normal samples (Fig. 1A). There were 12,525 upregulated genes and 6970 downregulated genes in Tumor vs. Normal (Fig. 1B). Tumor samples in TCGA-LUAD dataset were divided into high mRNAsi group (167 cases) and low mRNAsi group (333 cases) based on mRNAsi median value (0.4742294). we also found 9184 genes with increased expressions and 10,307 genes with decreased expressions in high group in comparison to low group (Fig. 1C). GO analysis in 19,491 DEGs showed regulation of hormone levels, axonogenesis, cell–substrate junction, DNA-binding transcription factor binding were enriched (Fig. S1A). KEGG analysis enriched to MAPK signaling pathway, human papillomavirus infection, neuroactive ligand-receptor interaction pathways (Fig. S1B).

Moreover, samples in high mRNAsi group had a less survival times than that in low mRNAsi group in TCGA-LUAD dataset (*p* = 0.042) (Fig. 1D), GSE31210 dataset (*p* = 0.009) (Fig. 1E), GSE50081 dataset (*p* = 0.043) (Fig. 1F). Differences analysis of clinical features between high- and low-mRNAsi group showed Gender, T.Stage, M.Stage and Stage had significantly various in TCGA-LUAD dataset (Fig. 1G), Stage and OS in GSE31210 dataset (Fig. 1H). but there were no differences in GSE50081 dataset (Fig. 1I).

### WGCNA

**Figure 2:**
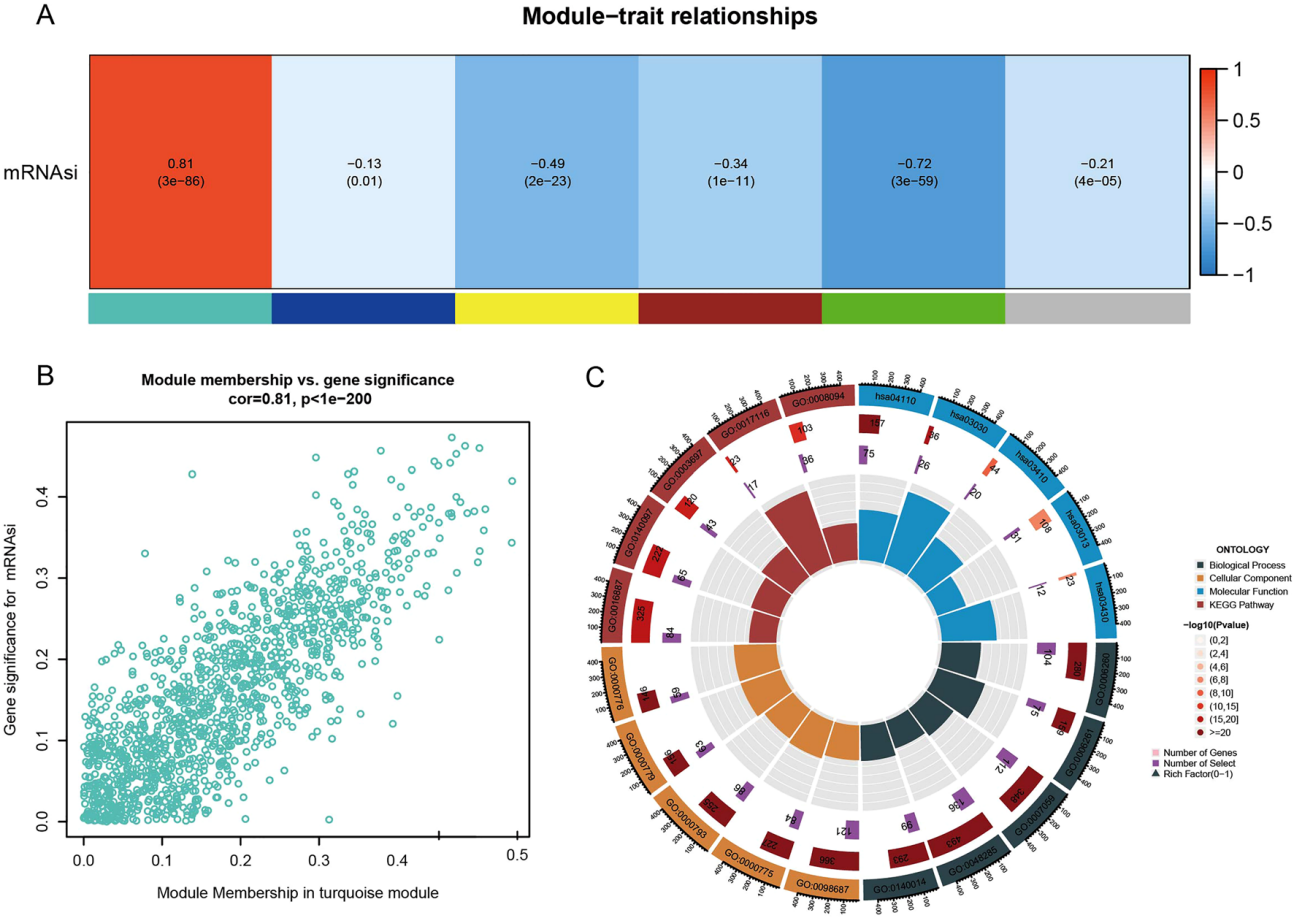
Weighted Co-Expression Network Analysis (WGCNA). (**A**) The module-trait relationships between mRNAsi and 6 modules. (**B**) Correlation analysis between gene significance for mRNAsi and module membership in turquoise module. (**C**) GO and KEGG analysis of genes in turquoise module.

To further screen mRNAsi related genes, WGCNA analysis was performed using mRNAsi score in TCGA-LUAD dataset. When soft threshold = 4 (Fig. S2A), 6 genes modules were determined (Fig. S2B). Correlation analysis between mRNAsi and 6 modules showed turquoise module was higher associated to mRNAsi (cor = 0.81, p = 3e−86) (Fig. 2A). A positive phenomenon was observed between gene significance for mRNAsi and module membership in turquoise module (cor = 0.81, p = 1e−200) (Fig. 2B). GO and KEGG analysis showed that the turquoise module genes were mainly enriched into many biological processes related to cell proliferation, such as DNA replication, mitosis, and organelle repair (Fig. 2C).

### Single cell analysis of CSCs

**Figure 3:**
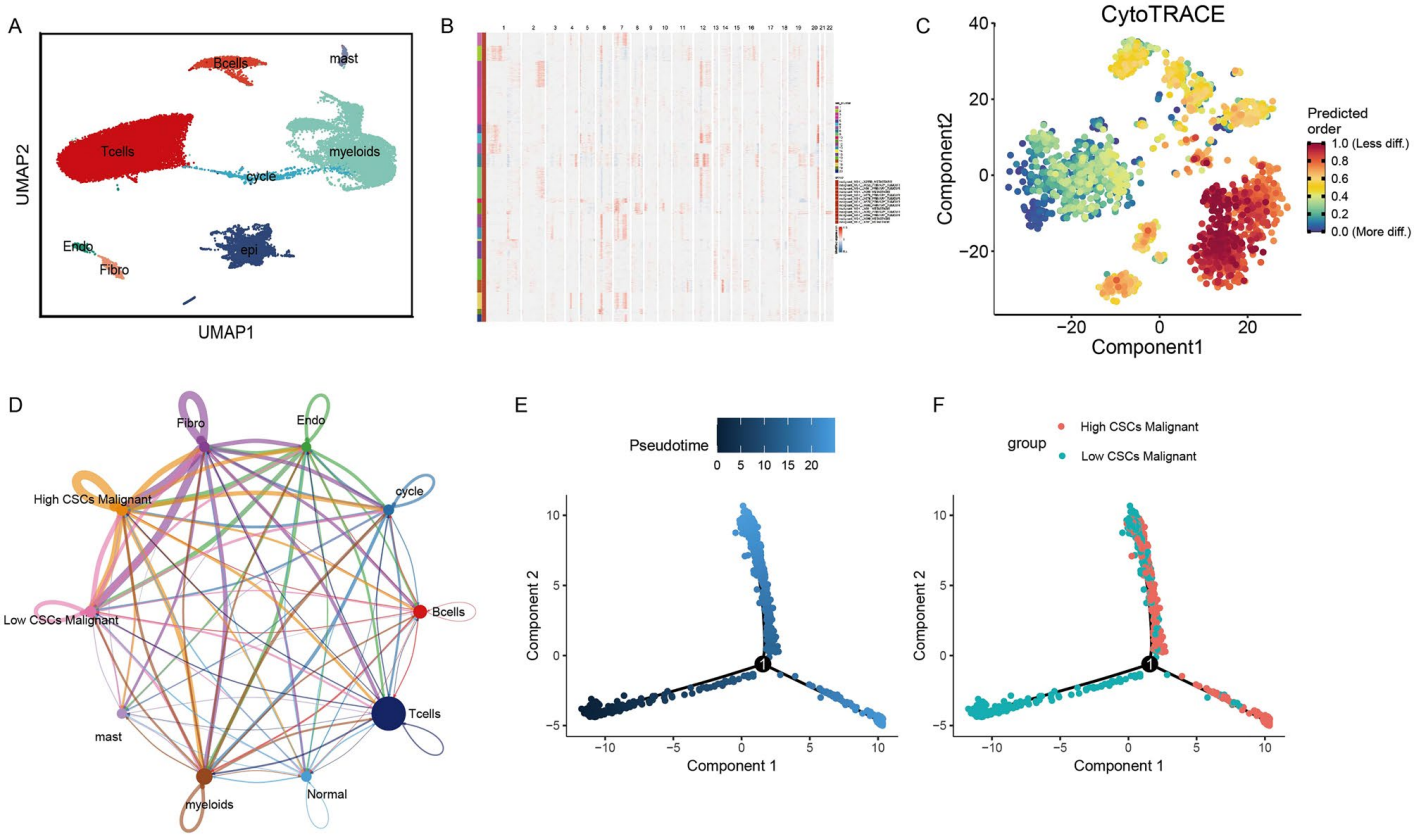
scRNA analysis in GSE123902 dataset. (**A**) 8 type cells were annotated in GSE123902 dataset. (**B**) Identification of malignant components in epithelial cells using Infercnv package. (**C**) Cytotrace package identified the high and low cancer stemness cell groups in malignant epithelial cells. (**D**) Cell communication analysis among high and low CSC malignant epithelial cells with other immune cells. (**E**,**F**) Pseudotemporal analysis of high and low CSC malignant epithelial cells.

Single cells in GSE123902 dataset were performed for dimension reduction and annotation analysis, and 8 cell subtypes were obtained (Fig. 3A). To identify the malignant tumor components in epithelial cells, we extracted epithelial cell subtypes for infercnv analysis, in which only cluster4,16 of epithelial cells were normal epithelial cells (Fig. 3B). To further clarify the stem differences in malignant epithelial cells, the malignant epithelial cells were extracted for CytoTRACE analysis, and high CSCs malignant epithelial cells and low CSCs malignant epithelial cells were defined based on the median CytoTRACE score (Fig. 3C). Cellcghat analysis indicated that high CSCs malignant epithelial cells had a more complex cellular communication with other cells (Fig. 3D). Pseudo-time series analysis was further used to explore the developmental trajectories of the high CSCs malignant epithelial cells and low CSCs malignant epithelial cells, and the results showed that malignant tumor cells developed from low CSCs to high CSCs malignant, indicating that there is a biological process of dedifferentiation with the progression of tumors (Fig. 3E,F).

### Key subtypes were identified by WGCNA

**Figure 4:**
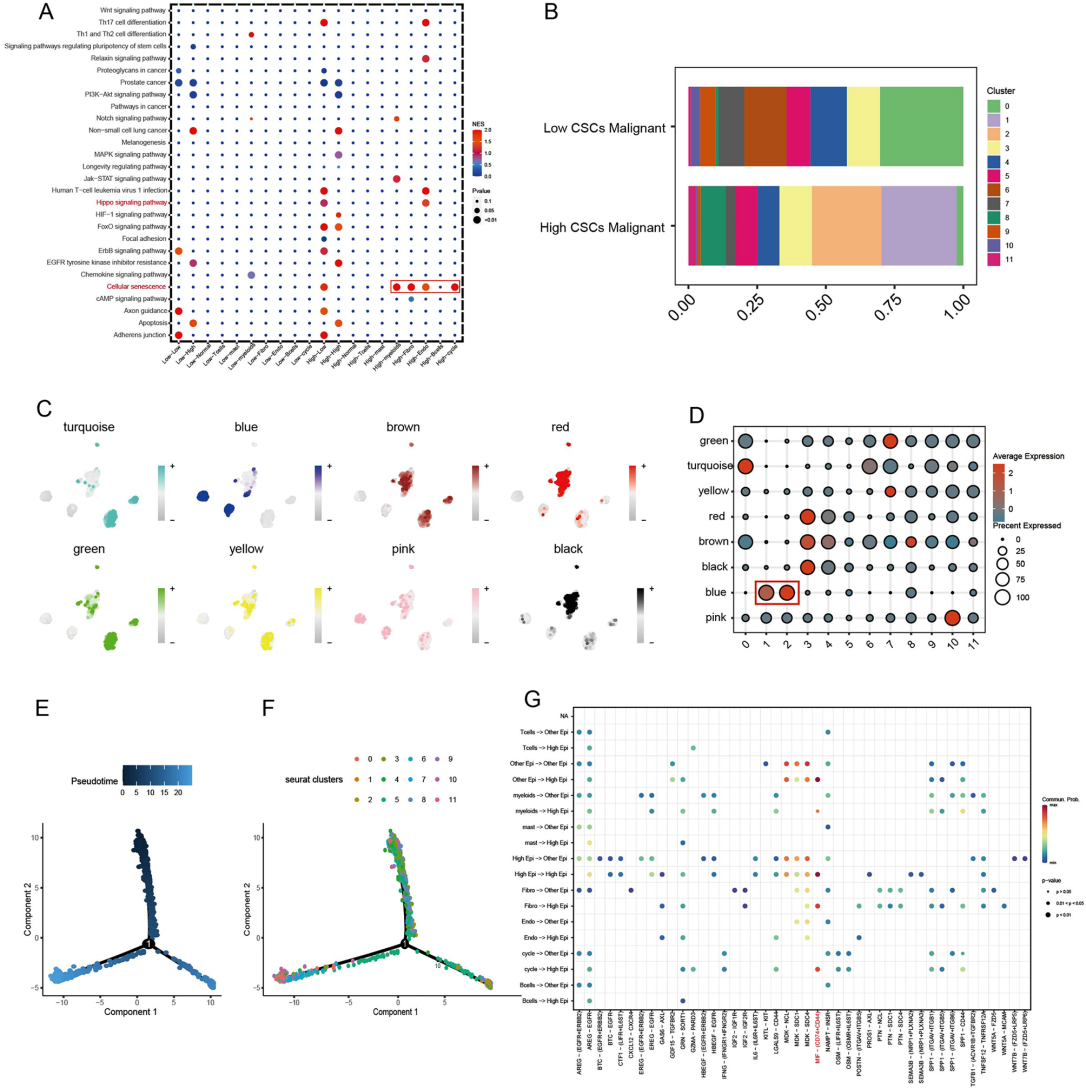
Hub cluster in CSC malignant epithelial cells through hdWGCNA. (**A**) CellCall analysis determined pathway differences between high and low CSC malignant epithelial cells. (**B**) The distribution of 12 clusters in high and low CSC malignant epithelial cells. (**C**,**D**) hdWGCNA found that blue modules were significantly enriched in cluster1 and custer2. (**E**,**F**) Pseudotemporal analysis of cluster1 and custer2. (**G**) Cellchat analysis showed communication differences of high CSC malignant epithelial cells.

CellCall analysis on low CSCs malignant and high CSCs malignant vs. other cell subtypes implied that Cellular senescence pathway and Hippo signaling pathway were main difference pathways (Fig. 4A). Moreover, cluster1, 2 only existed in high CSCs malignant group (Fig. 4B). WGCNA analysis indicated that bule module enriched in cluster1, 2 (Fig. 4C,D). The pseudo-time series analysis showed that cluster1 and cluster2 were concentrated at the end of differentiation trajectory (Fig. 4E,F), which was consistent with our previous studies and further verified the biological characteristics of dry dedifferentiation of LUAD. cluster1 and cluster2 were defined as High epi group, and MIF-(CD74 + CD44) were increased in High epi group (Fig. 4G).

### Construction of prognosis model based on machine learning

**Figure 5:**
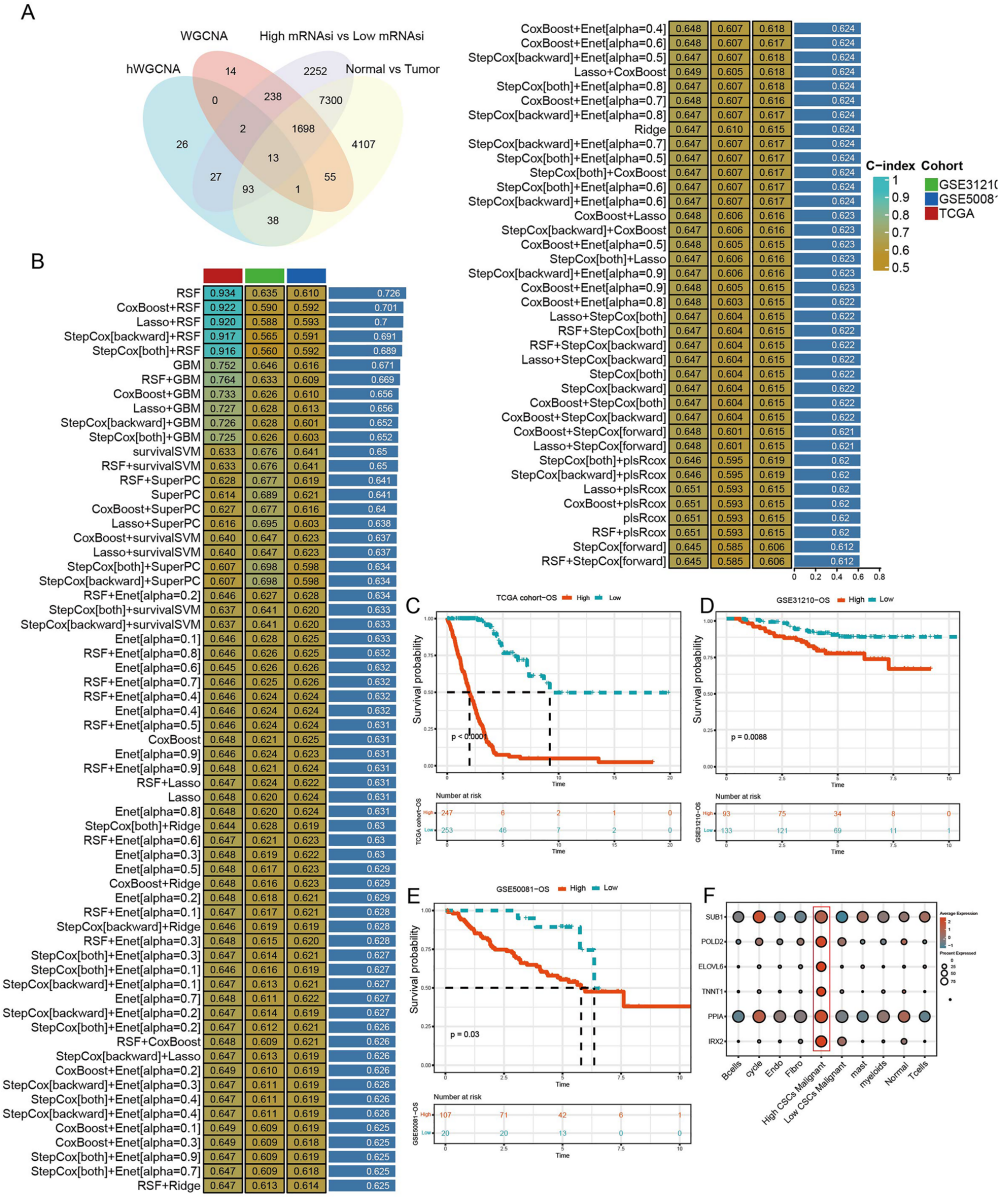
Construction of prognosis model. (**A**) Venn diagram of differentially expressed genes. (**B**) 101 prognostic prediction models were built by machine learning constructs. (**C**–**E**) KM survival curve of prognosis model in TCGA dataset, GSE31210 dataset and GSE50081 dataset. (**F**) Hub gene localization in single cell subpopulation.

13 key genes were obtained by intersection of top200 genes in hdWGCNA, genes in turquoise module, DEGs in high vs. low mRNAsi group and DEGs in tumor vs. normal samples (Fig. 5A). In TCGA-LUAD dataset, 101 prognosis models were detected by LOOCV frame and c index of 101 models were calculated in TCGA-LUAD dataset, GSE50081 dataset, GSE3210 dataset. Among which, average c index was highest (0.701) of CoxBoost + RFS model (Fig. 5B). 6 hub genes (SUB1, POLD2, ELOVL6, TNNT1, PPIA, IRX2) were screened. KM survival analysis demonstrated that samples in high group had a less survival time in TCGA-LUAD dataset, GSE50081 dataset, GSE3210 dataset (Fig. 5C–E). In addition, based on single-cell data, hub gene positioning was further defined, and the results showed that 6 genes were significantly highly expressed in high CSCs malignant epithelial cells (Fig. 5F).

### Immune microenvironment landscape analysis basis on prognosis model

**Figure 6:**
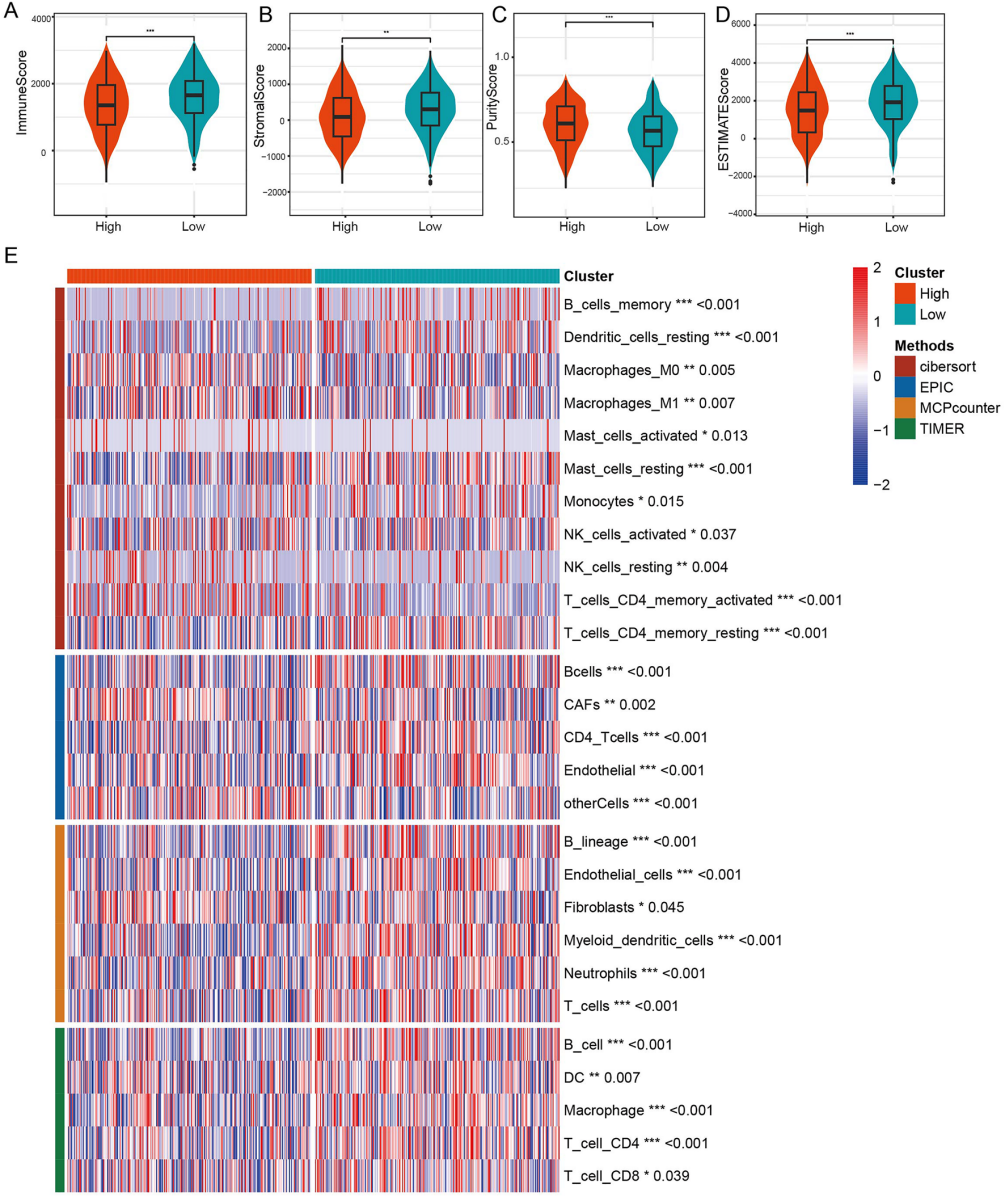
Immune microenvironment analysis. (**A**) ImmuneScore differences between high- and low-risk group. (**B**) StromalScore differences between high- and low-risk group. (**C**) PurityScore differences between high- and low-risk group. (**D**) ESTIMATEScore differences between high- and low-risk group. (**E**) CIBROSORT, EPIC, MCP-counter, TIMER analysis between high- and low- risk group.

ESTIMATE analysis showed that ImmunScore, StromalScore and ESTIMATEScore were enhanced in high group that those in low group (Fig. 6A–C). PurityScore was decreased in high group, indicating a higher tumor malignancy (Fig. 6D). Subsequently, CIBROSORT, EPIC, MCP-counter and TIMER analyses also verified that there was significant immunosuppression in the high group (Fig. 6E).

### Relationship between 6 hub genes and immunity, pathways immunity, pathway

**Figure 7:**
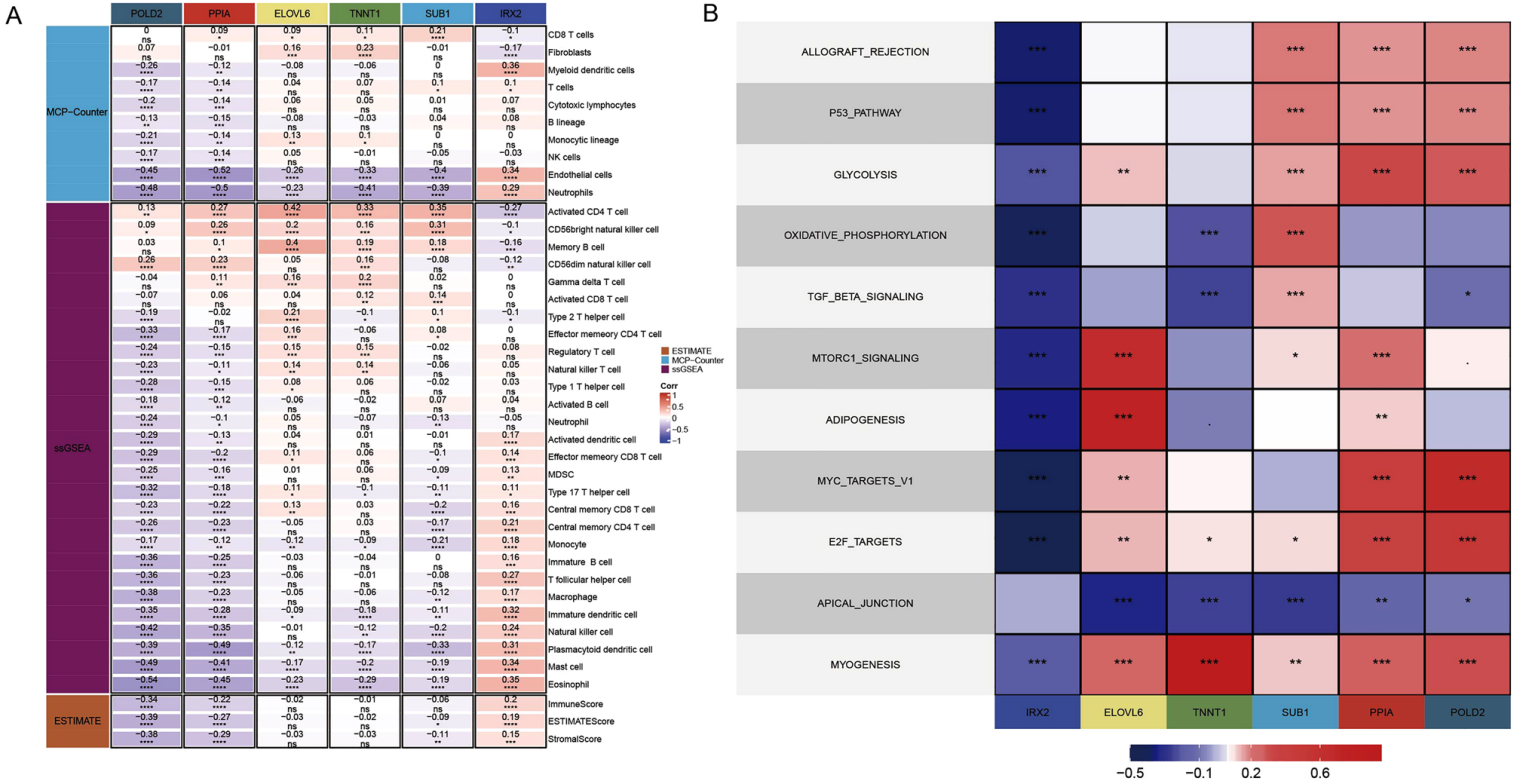
Correlation analysis between hub genes and immunity/pathways. (**A**) Correlation analysis between hub genes and immunity in TCGA dataset. (**B**) Correlation analysis between hub genes and pathways in TCGA dataset.

The ESTIMATE and MCP-counter methods were used to evaluate the immune scores of samples from GSE75214 dataset, and the ssGSEA method was used to evaluate the scores of 28 immune cells corresponding to each sample. Next, the Pearson correlations between 6 hub genes and these immune scores were calculated and visualized, among which, except SUB1 and IRX2, other 4 genes were negatively correlated with major immune killer cells (Fig. 7A). The Pearson correlations between 11 pathways scores and 6 hub genes indicated that all genes were negatively to APICAL_JUNCTION pathway (Fig. 7B).

### Predictive analysis of chemotherapy drugs and immunotherapy

**Figure 8:**
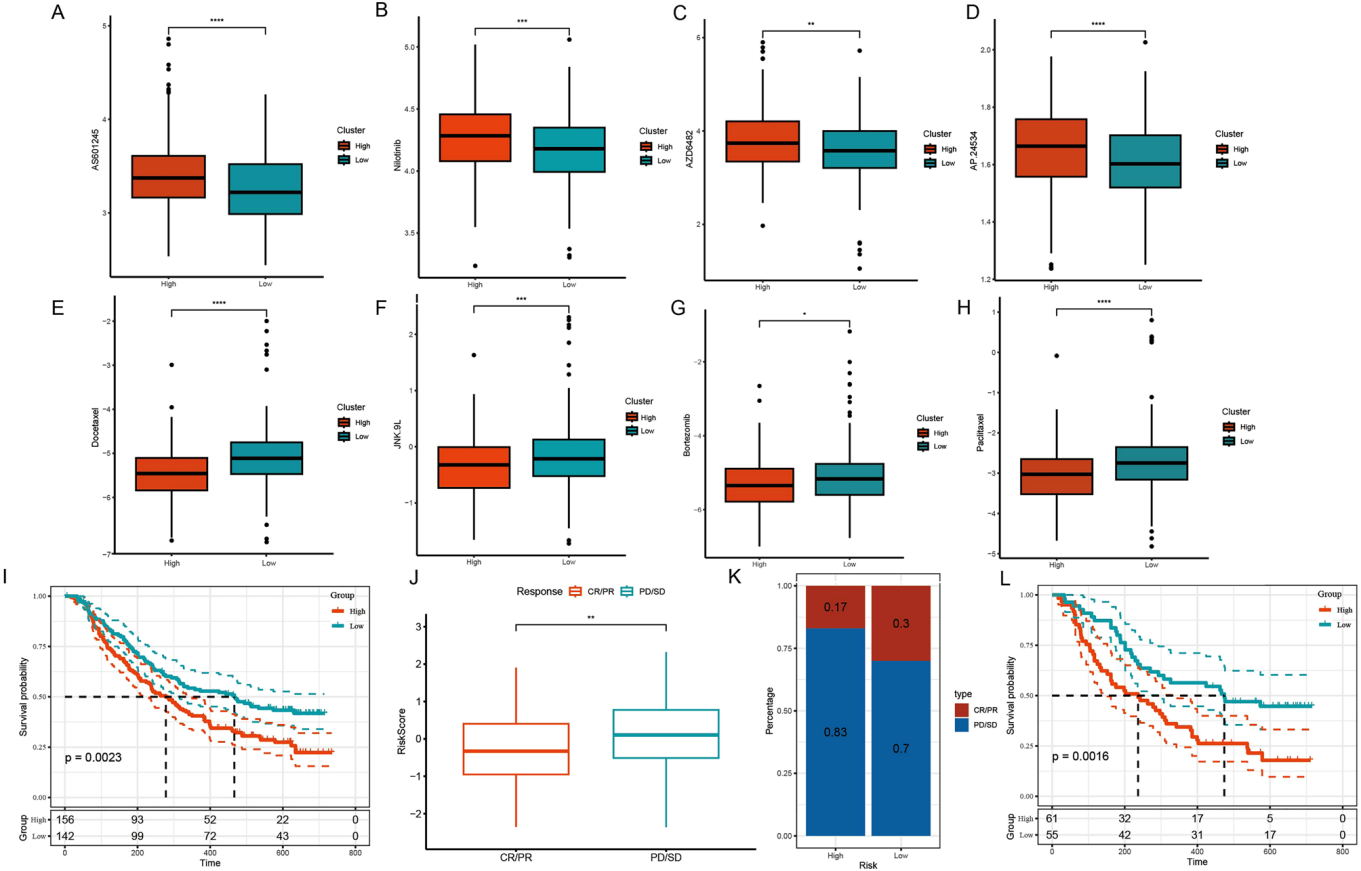
Prognostic model to predict the efficacy of chemotherapy drugs and immunotherapy. (**A**–**H**) IC50 differences of chemotherapy drugs between high- and low- risk groups. (**I**) The survival times in high-risk group was worse in IMvigor210 dataset. (**J**) Differences in RiskScore between CR/PR and PD/SD responses in the IMvigor210 cohort. (**K**) Distribution of immunotherapy response between high- and low- risk groups in the IMvigor210 cohort. (**L**) The high-risk group of III-IV patients had a worse prognosis.

In TCGA-LUAD dataset, drug sensitivity prediction analysis of prognostic model showed low-risk group was benefit from AS601245, Nilotinib, AZD6482, AP.24534 (Fig. 8A–D). The high-risk group showed better sensitivity to Docetaxel, JNK.9L, Bortezomib, and Paclitaxel (Fig. 8E–H), which provided a direction for later treatment. In IMvigor210 dataset, patients in the high-risk group treated with PD-L1 had a worse prognosis (*p* = 0.0023, Fig. 8I). RiskScore of SD/PD samples were higher than that in CR/PR samples (Fig. 8J). High-risk group had more PD/SD samples (Fig. 8K). In stage III-IV patients, the high-risk group had a worse prognosis (*p* = 0.0016, Fig. 8L).

## Discussion

Today, CSCs are seen as drivers of tumor establishment and growth and are often associated with aggressive, heterogeneous, and treatment-resistant tumors^11–14^. In colon cancer, recent studies in mice have shown that even differentiated intestinal epithelial cells may act as potential CSCS^15^. Epithelial cell adhesion molecules (EpCAM, CD326) are expressed on CSCS of multiple tumor types, including colon and liver cancer^16,17^. CSC is found in almost all solid tumors^18^. Motivated by these observations, In LUAD, we hypothesize that CSC is associated with malignant epithelial cells. Using transcriptome data of LUAD, it was found that tumor samples had higher mRNAsi, and samples in the high-dry group had worse prognosis. WGCNA analysis showed that turquoise modules were highly correlated with mRNAsi and were associated with biological processes such as cell proliferation. scRNA analysis identified 12 clusters of epithelial cells, and malignant tumor cells developed from Low CSCs to High CSCs. hdWGCNA indicated that blue modules are significantly enriched in cluster1 and Cluster2, and there are differentiation trajectories at the end.

Cellcall analyzed the pathway differences between High CSCs malignant and low CSCs malignant and other cell subpopulations, the results showed that cellular senescence and Hippo signaling pathway were the major difference pathways. Dysregulation of the Hippo signaling pathway is associated with cancer progression, including aberrant expression and activity of YAPs and TAZs, and deficiencies in large tumor suppressor kinase 1/2 (LATS1/2)^19–21^. The role of YAP/TAZ in cancer stem cells and tumour recurrence is supported by recent evidence^22,23^. In addition, Hippo signaling pathway is mainly concentrated in endothelial cells, which may be closely related to angiogenic mimicry^24,25^. A CSC-like phenotype can be acquired by epithelial-mesenchymal transition (EMT) programs or by escaping from senescence^26^. These results suggest that Cellular senescence and Hippo signaling pathway may be involved in the deterioration of epithelial cells.

A prognostic model was constructed based on machine learning and six key genes (SUB1, POLD2, ELOVL6, TNNT1, PPIA, IRX2) were screened. Several studies have shown that POLD2 is aberrantly expressed in multiple cancers, including ovarian carcinoma^27^ and glioblastoma^28^. Accumulating evidence has demonstrated that ELOVL6 is high-expressed and serves as a negative clinical predictor in a plenty of carcinomas^29,30^. TNNT1 has been reported to contribute to the progression of colorectal cancer^31^ and breast cancer^32^, colon adenocarcinoma^33^. PPIA has been implicated in a broad range of pathological processes, including inflammatory diseases, aging and the progression of cancer metastasis^34^. Previous studies have demonstrated that overexpression of PPIA plays key roles in different types of cancer, including hepatocellular carcinoma, lung cancer, pancreatic cancer, breast cancer, colorectal cancer, squamous cell carcinoma and melanoma^35^.

This study inevitably has some limitations. Firstly, our research data came from a public database, not our own. Although the validation set is sufficient to support the conclusions of our study, further validation of the prognostic and therapeutic effects of this model from our own center using a large sample size is needed in the future. Then, further functional experiments will be required to elucidate the biological mechanisms of these genes in lung adenocarcinoma stemness and TME landscape, and to determine whether they could be targeted to improve the effectiveness of immunotherapies and chemotherapies, Thirdly, the stem cell dataset (PCBC dataset) of prostate cancer was applied to lung adenocarcinoma, which does not have pluripotent stem cell data sets, is worthy of further study and exploration of its appropriateness and universality.

In summary, analysis of both scRNA-seq and bulk RNA-seq in LUAD samples showed the CSC characteristics of the cancer transformation process from epithelial cell. Based on differentially correlated CSC-related genes, we constructed prognostic and immune-related models. We suggested that our stemness model has future clinical implications for prognostic evaluation and may help clinicians to select likely responders for prioritised use of current immune checkpoint inhibitors.

## Methods

### Data acquisition and processing

The GSE123902 single-cell dataset was downloaded from the Gene Expression Omnibus (GEO) database (https://www.ncbi.nlm.nih.gov/geo), and samples were obtained from 8 patients with primary lung adenocarcinoma, 3 patients with brain metastases, 1 patient with bone metastases, 1 patient with adrenal metastases, and 4 patients with normal lung tissue, a total of 41,384 cells were obtained. PercentageFeatureSet function R package Seurat (https://satijalab.org/seurat/) is used to calculate the percentage of mitochondria, ribosomes and erythrocytes. Cells were selected with more than 300 expressed genes, less than 15% mitochondrial gene expression and less than 1% erythrocyte gene proportion. Then, the combined scRNA-seq data was normalized, and the Top 2000 highly variable genes were found by FindVariableFeature function R package Seurat, and the ScaleData function R package Seurat was used to scale all genes, and the RunPCA function was used to reduce the dimensionality of the Top 2000 highly variable genes selected. Batch correction is then performed using the harmony algorithm. The “FindNeighbors” and “FindCluster” functions (resolution = 0.8) R package Seurat are used to cluster cells when dim = 20. Next, we use the RunUMAP method for further dimensionality reduction. Finally, we screened the marker genes (Table 1) of subpopulation using the FindAllMarkers function, annotated and visualized them using references^36^ and cellmarker2.0^37^. Tumor cell identification was performed using the inferCNV package. and mimetic time-series analysis of tumor cell subpopulations using the Monocle2 package^38^.

**Table 1:** Marker genes of immune cells.

Cells	Marker genes
T cell	PTPRC, CD3′, CD3E, CD4, CD8A
B cell	CD19, CD79A, MS4A1
Mast cell	IGHG1, MZB1, SDC1
Myeloid cell	C1QA, C1QB, S100A9, S100A8, MMP1
Fibroblast	FGF7, MME, DCN, LUM, GSN
Endothelial cell	PECAM1, VWF
Epithelial cell	EPCAM, KRT19, KRT7

In addition, transcriptome data of LUAD and pancarcinoma with survival information were obtained from the University of California Santa Cruz (UCSC) database (https://xenabrowser.net/). And the GSE312104^39^, GSE500815^40^ datasets were downloaded from the GEO database for subsequent transcriptome level validation. All the data required for this study can be searched through public databases. According to the group information, DEseq2 R package^41^ was used for differential expression analysis under adj.pvalue < 0.05, |log2FC| > 1. The intersection of the above differential expression genes will be taken as the next step.

### Tumor stemness calculation

mRNA expression based stemness index (mRNAsi) reflects the gene expression characteristics of stem cells. mRNAsi developed predictive models for multipotent stem cell samples (ESC and IPSC) from the PCBC dataset by using One Class Linear Regression (OCLR)^42^. Then the model is applied to the GEO datasets to calculate the stemness score of each sample and finally evaluate the stemness degree of each sample, which divided the samples into high and low stemness groups. DEseq2 R package was used to analyze DEGs between samples with high mRNAsi and low mRNAsi with condition was p.al < 0.05, |log2FC| > 1. The ComplexHeatmap package^43^ and ggplot2 package were respectively used to draw heatmaps and volcano maps.

### Functional enrichment analysis

Gene Ontology (GO) analysis is a common method to conduct large-scale functional enrichment studies, including biological process (BP), molecular function (MF), and cellular component (CC). The Kyoto Encyclopedia of Genes and Genomes (KEGG)^44–46^ is a widely used database for storing information about genomes, biological pathways, diseases and drugs. GO annotation analysis and KEGG pathway enrichment analysis of differential genes were performed using clusterProfiler R software package^47^, and the critical value of FDR p < 0.05 was considered to be statistically significant.

### Weighted co-expression network analysis (WGCNA)

Weighted Co-Expression Network Analysis (WGCNA)^48^ is a systems biology method used to describe patterns of genetic associations between different samples. The samples with missing values and discrete samples are deleted. Selecting the optimal soft threshold β (β = 4) was selected to construct a WGCNA. In addition, the weighted adjacency matrix is transformed into a topological overlap matrix (TOM) to estimate the connectivity of the network. Then, the hierarchical clustering method is used to construct a clustering tree to determine that the module size is set to 80, and the threshold of similarity module merging is set to 0.35. Later, Pearson’s correlation between module eigengene and mRNAsi was performed to obtain mRNAsi related module.

### CytoTRACE

CytoTRACE^49^ presents a new framework for calculating cell differentiation capacity that utilizes gene counting to significantly improve cell differentiation at the single-cell level. Unlike most existing lineage trajectory analysis methods, CytoTRACE can predict relative states and directions of differentiation in a way that is independent of the presence of continuous developmental processes in a particular time scale or data, and independent of the presence or absence of continuous developmental processes in a particular time scale or data. Herein, CytoTRACE is used to calculate cell stemness score in tumor epithelium, and the epithelium is divided into High CSCs epi group and Low CSCs epi group according to the median stemness score.

### High dimensional WGCNA

High dimensional WGCNA (hdWGCNA) was used for WGCNA in single-cell RNA-seq. After set the threshold of scale-free topology model fit as > 0.85, soft threshold was selected as 4 for the best connectivity. Based on TOM, average-linkage hierarchical clustering method was used to cluster genes under the height = 0.25, deepSplit = 2, and minModuleSize = 300 standards. Pearson’s correlation was conduct between gene module and mRNAsi.

### Cell communication

CellCall^50^ is a toolkit that collects ligand-receptor-transcription factor (L-R-TF) axis data sets based on the KEGG pathway to infer intercellular communication networks and internal regulatory signals by integrating intracellular and intercellular signals. We used CellCall to further clarify the specific pathway between the high-low rating group and other SCLC subtypes.

R package CellChat (V1.6.0)^51^ used the data of single cells and our cell classification for cell communication analysis, used the built-in CellChat CellChatDB. Human as a reference to analyze the interactions between cells, and analyzed the relationship between 32 pathways.

### Correlation analysis between key genes and immunity/pathways

ESTIMATE algorithm^52^, obtaining public website (https://sourceforge.net/projects/estimateproject/), used to estimate StromalScore and ImmuneScore based on specific biomarkers associated with stromal cell and immune cell infiltration in tumor samples. Then the Pearson correlation of key genes to them was calculated.

The MCP-counter^53^ method enables robust quantification of the absolute abundance of eight immune cells and two stromal cell populations (T cells, CD8 T cells, Cytotoxic lymphocytes, B lineage, NK) cells, Monocytic lineage, Myeloid dendritic cells, Neutrophils) in heterogeneous tissues from transcriptome data. Then the Pearson correlation of key genes to them was calculated.

Gene set variation analysis (GSVA)^54^ is a nonparametric, unsupervised gene-set enrichment method that estimates pathway or hallmmarker scores based on transcriptome data. The ssGSEA method in R package GSVA was used to obtain the genes of 28 kinds of immune cells from the literature and calculate the scores.

In addition, 50 HALLKMARK pathways in h.all.v7.5.symbols.gmt were obtained from the GSEA website, and the pathway scores of samples were calculated using ssGSEA method, and then the correlation between key genes and them was calculated.

### Construction and validation of prognostic model

To develop a model with high accuracy and stable performance, we integrated 10 machine learning algorithms and 101 algorithm combinations. The comprehensive algorithms include random survival forest (RSF), Elastic network (Enet), Lasso, Ridge, stepwise Cox, CoxBoost, Cox Partial least squares regression (plsRcox), supervised Principal Component (SuperPC), generalized enhanced regression model (GBM), and survival support vector machine (Survival-SVM). The signature generation procedure was as follows: (a) univariate Cox regression identified prognostic related differentially expressed genes in the TCGA-LUAD cohort; (b) The prognostic genes were then combined with 101 algorithms to fit the prediction model based on the leave-one cross-validation (LOOCV) framework in the TCGA-LUAD cohort; (c) All models were detected in two validation datasets (GSE31210, GSE50081); (d) For each model, the Harrell Consistency Index (C-index) is calculated on all validation datasets, and the model with the highest average C-index is considered to be the optimal. The survminer R package was used to plot the survival curve of the high- and low- risk group.

### Statistical analysis

Statistical analyses were performed using R version 3.4.0. P values were two-sided, and P < 0.05 was considered statistically significant. The pRRophetic package^55^ was used to predict chemotherapy drugs in the high-low risk group.

### Supplementary Information

Supplementary Figure S1.

Supplementary Figure S2.

## Data Availability

The datasets generated during and/or analysed during the current study are available from the corresponding author on reasonable request.
