# Supplementary figures and images for: scRNA-seq revealed high stemness epithelial malignant cell clusters and prognostic models of lung adenocarcinoma
Source: Sci Rep. 2024 Feb 14;14:3709. doi: 10.1038/s41598-024-54135-4 (PMC10867035; doi:10.1038/s41598-024-54135-4)

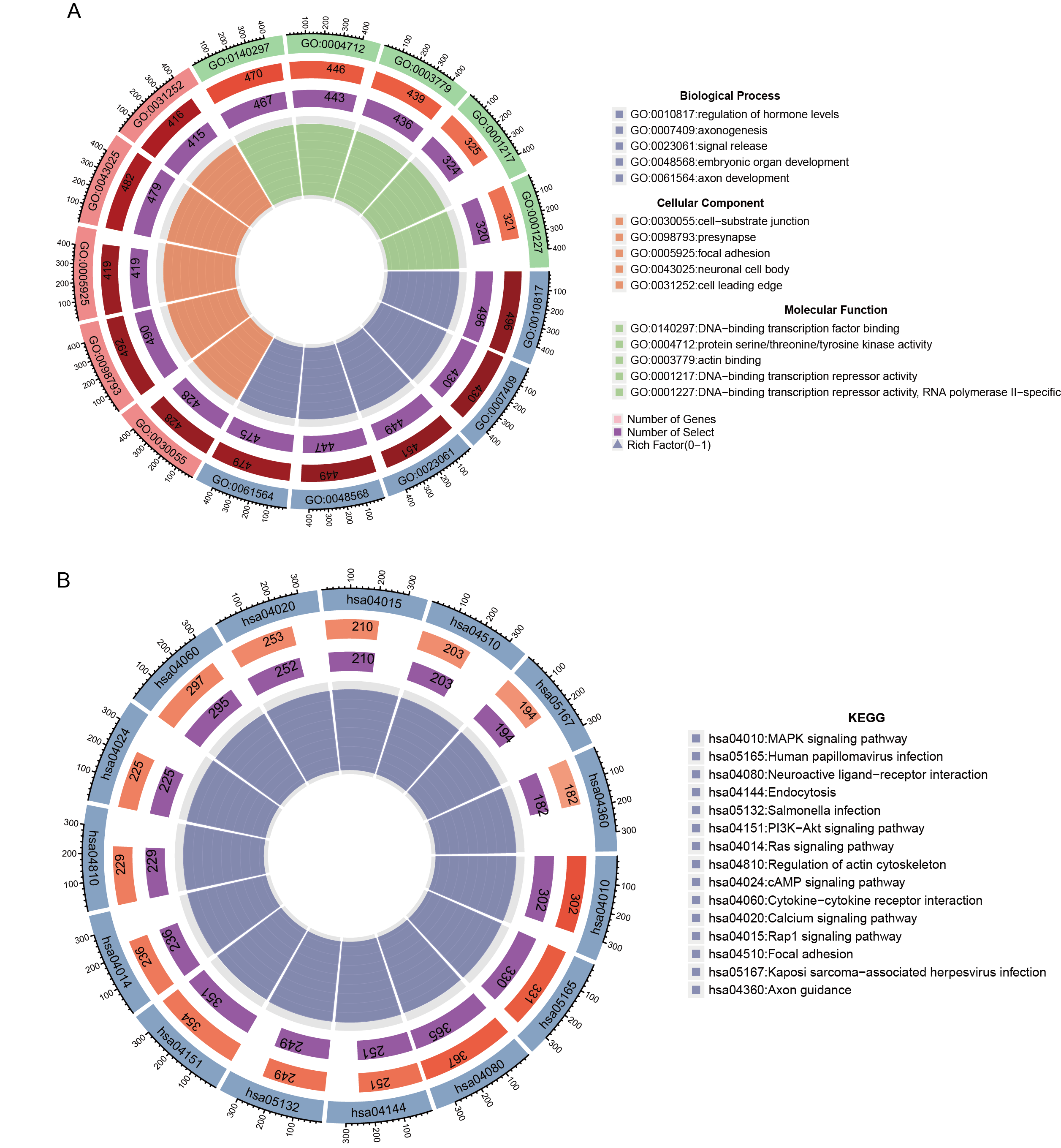

Supplement: Supplementary file 1 — Supplementary Figure S1. [file 41598_2024_54135_MOESM1_ESM.png]

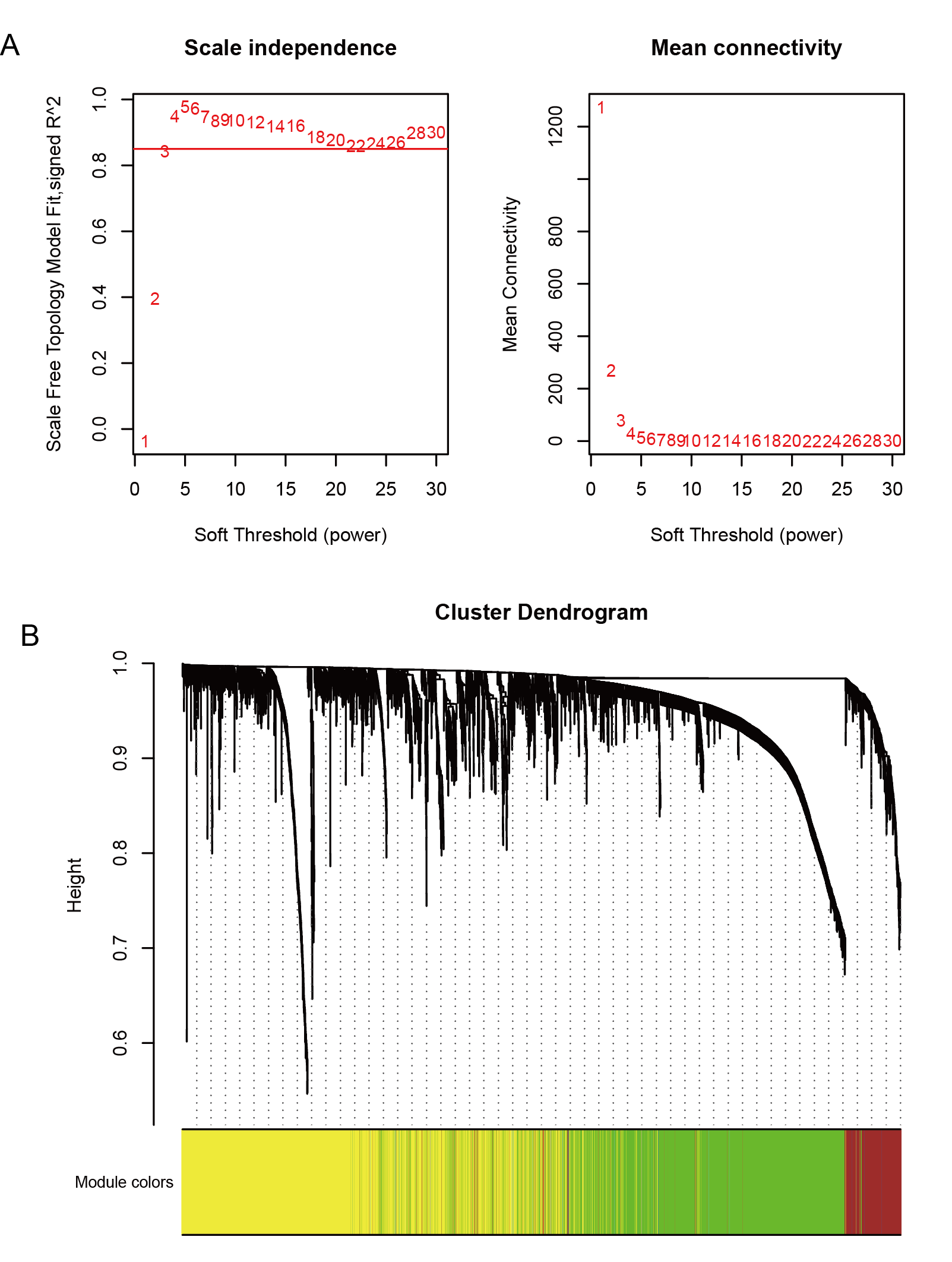

Supplement: Supplementary file 2 — Supplementary Figure S2. [file 41598_2024_54135_MOESM2_ESM.png]
